# Recent Advances in the Synthesis of Cyclic Sulfoximines via C–H Bond Activation

**DOI:** 10.3390/molecules28031367

**Published:** 2023-02-01

**Authors:** Bingren Wang, Xiayu Liang, Qingle Zeng

**Affiliations:** State Key Laboratory of Geohazard Prevention and Geoenvironment Protection, College of Materials, Chemistry & Chemical Engineering, Chengdu University of Technology, Chengdu 610059, China

**Keywords:** cyclic sulfoximines, C–H activation, cyclization, synthesis, synthetic methods

## Abstract

Sulfoximines, a ubiquitous class of structural motifs, are widely present in bioactive molecules and functional materials that have received considerable attention from modern organic chemistry, pharmaceutical industries, and materials science. Sulfoximines have proved to be an effective directing group for C–H functionalization which was widely investigated for the synthesis of cyclic sulfoximines. Within the last decade, great progress has been achieved in the synthesis of cyclic sulfoximines. Thus, this review highlights the recent advances in the synthesis of cyclic sulfoximines via the C–H activation strategy and is classified based on the substrate types.

## 1. Introduction

**Figure 1:**
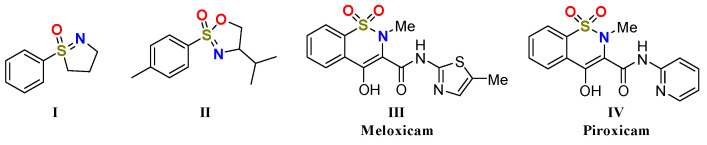
Structures of bioactive cyclic sulfoximines and analogues.

In the field of cancer treatment, sulfoximine, which was found as a toxic compound in the 1940s, is now called Rising Star due to its unique significance in recent drug discovery [1,2,3]. Since sulfoximines are the isosteres of sulfones with a mono-aza nitrogen atom [4], they have special properties, such as the sulfur atom with an optical rotation property [5,6], the S=N bond with the double bond property, and nitrogen with weak nucleophilicity [7]. Sulfoximines have been widely applied in asymmetric catalysis, synthesis of bioactive molecules, and modern pharmaceutical and agrichemical industries [8]. Over the past decades, cyclic sulfoximines, as an important kind of sulfoximine derivatives, attracted a great deal of attention for chemists. Due to their outstanding biological properties, a number of molecules with potential medical application values appeared [9], for example, five-membered cycle **I** which performed well under in vitro pharmacokinetic studies [10], and motif **II** which could be transformed to potential scaffolds for peptide mimetics [11]. Furthermore, analogues which also have the S=O bond and S–N bond, such as 1,2-benzothiazine 1,1-dioxides **III** and **IV**, were found to be anti-inflammatory drugs (Figure 1) [12]. 

It was once overlooked, but it has been re-emphasized recently that sulfoximine derivatives possess desirable properties as promising drug candidates. Additionally, C–H activation of sulfoximines has been widely considered to be an efficient method to construct complex scaffolds with potential bioactivities, especially since Bolm discovered the first efficient strategy of Rh-catalyzed annulation of sulfoximines and alkynes in 2013 [13]. In addition, methyl phenyl sulfoximine (MPS) was proved to have better coordination adaptability to the transition metal center in C–H activation steps than some other nitrogen hetero groups [14]. In a word, the cyclic sulfoximines exhibit great values, especially in the medicinal field. 

**Figure 2:**
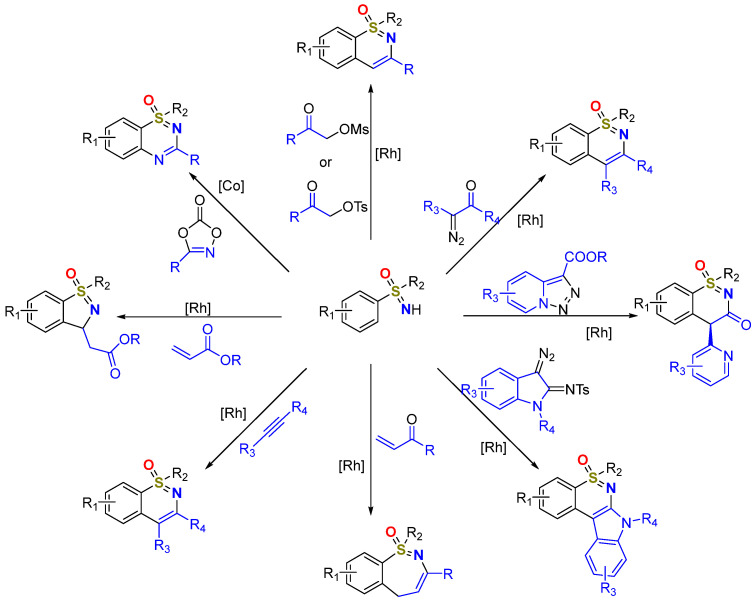
The main strategies towards cyclic sulfoximines synthesis.

With the above concepts in mind, and based on our long-term studies on the synthesis of sulfoximines [15,16,17,18,19], we concentrate on the advances in the synthesis of cyclic sulfoximines via C–H bond activation since 2018 in this review. Additionally, the previously developed main approaches towards cyclic sulfoximines from sulfoximines are shown in Figure 2. It was worth noting that the Co-catalyzed strategy was difficult to conduct smoothly but breakthroughs were also made in this area recently [13,20,21,22,23,24,25,26,27].

## 2. Synthesis of Cyclic Sulfoximines via Intermolecular C–H Activation

### 2.1. Metal Carbenoids as Coupling Partners

#### 2.1.1. Diazo Compounds as Carbene Precursors

**Scheme 1:**
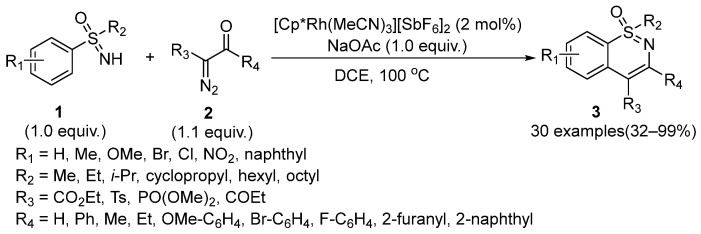
Rhodium-catalyzed annulation reactions for the synthesis of 1,2-benzothiazines.

In 2015, Bolm and coworkers first described a Rh(III)-catalyzed intermolecular annulation between sulfoximines **1** and diazo compounds **2** for the synthesis of 1,2-benzothiazine products **3** (Scheme 1) through the domino C–H activation/cyclization/condensation pathway [21]. DCE was proved to be the optimal solvent and the yield of desired products reached up to 99% in the presence of NaOAc (1.0 equiv) as a base. Moreover, this kind of C–H activation featured wide substitution tolerance and afforded the desired adducts with low to excellent yields (32–99%).

**Scheme 2:**
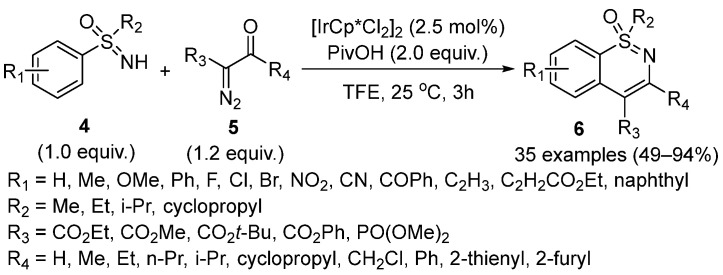
Ir(III)-catalyzed C–H functionalization of sulfoximines with α-diazocarbonyl compounds.

In 2018, the Ir(III)-catalyzed C–H functionalization of sulfoximines **4** with α-diazocarbonyl compounds **5** (Scheme 2) was first developed by Pawar and coworkers [28]. Furthermore, this C–H functionalization system was compatible with a broad scope of sulfoximines including electron-donating substituents, halogen substituents, and olefin motifs. However, the presence of strong electron-withdrawing groups such as NO_2_ and CN significantly resulted in lower yields. Moreover, diazo compounds bearing various substituents were also tolerated in this protocol.

**Scheme 3:**
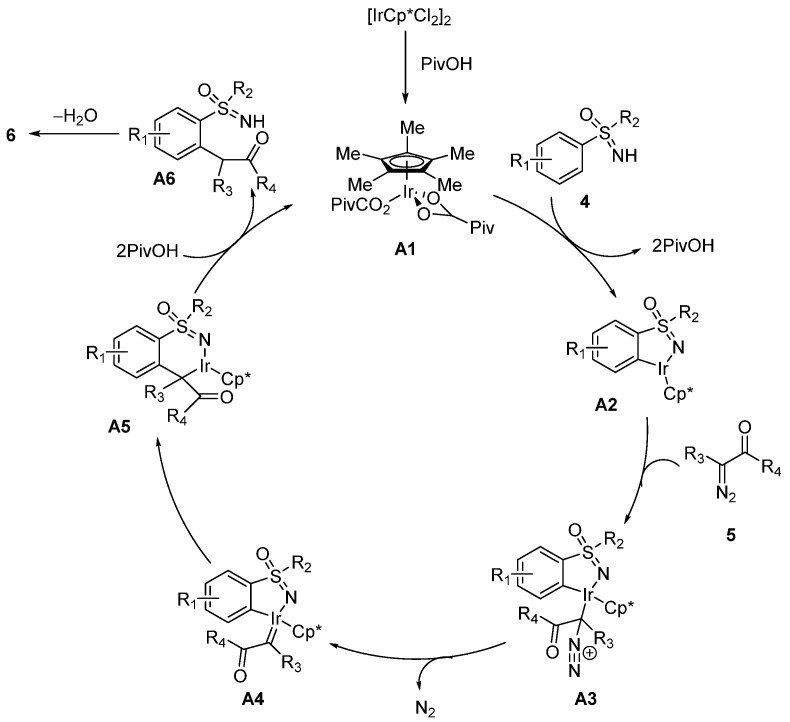
Proposed mechanism of the formation of 1,2-benzothiazines in the Ir(III) catalytic system.

The plausible mechanism (Scheme 3) involves the following steps: firstly, neutral Ir(III) species **A1** with catalytic activity formed by [Cp*IrCl_2_]_2_ reacts with pivalic acid. Next, NH-sulfoximines **4** coordinate to the metal center to generate five-membered iridacycle **A2** via deprotonation and metallization. Subsequently, **A2** is coordinated with diazo precursors **5** and the loss of N_2_ generates carbene species **A4**. Then, the alkylated migratory insertion takes place, and subsequent protonolysis affords alkylated product **A6** and Ir(III) species **A1**. Finally, **A6** undergoes the acid-promoted cyclization/dehydration process to give the expected product **6**.

**Scheme 4:**
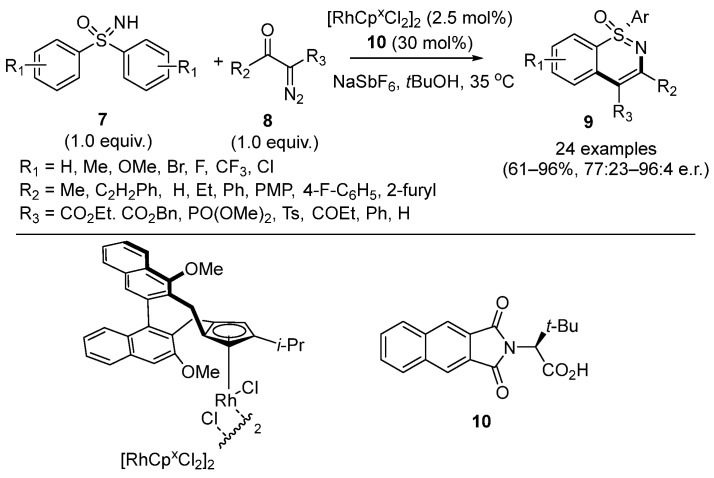
Synthesis of chiral-at-sulfur 1,2-benzothiazines via C–H functionalization of sulfoximines.

In the same year, Cramer’s group reported the enantioselective [4+2] annulative coupling reaction of diaryl sulfoximines **7** and acyl diazo compounds **8** for the synthesis of chiral-at-sulfur 1,2-benzothiazines **9** catalyzed by [RhCp*Cl_2_]_2_ and N-protected amino acid **10** (Scheme 4) [29]. Under the optimal condition, various substituted cyclic products were given with good to excellent yields (61–95%) and moderate to excellent enantioselectivities (77:23–96:4 e.r.). Moreover, it should be highlighted that the addition of polyfluoro-substituted alcohols such as TFE and HFIP as the solvents had a profound effect on the enantioselectivity for this reaction which gave the opposite main enantiomeric products.

**Scheme 5:**
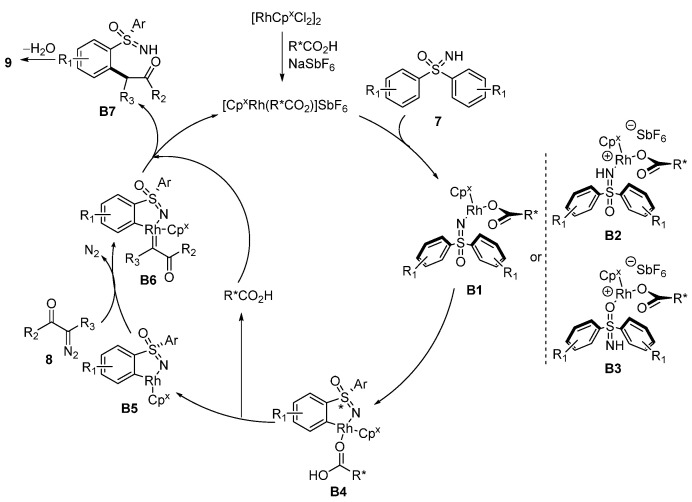
Proposed mechanism of Rh-catalyzed chiral-at-sulfur 1,2-benzothiazines formation.

A plausible mechanism is shown in Scheme 5, which is similar to the achiral version reported by Bolm. At first, sulfoximines **7** coordinate to the Rh(III) center to produce **B1** (possibly **B2** or **B3**), and initiate the enantiodetermining *ortho*-C–H activation step by a concerted metalation deprotonation pathway to give intermediates **B4**. Then, five-membered cyclic rhodium complexes **B5** are formed by the auxiliary of the N-protected amino acid. Subsequently, carbenoid intermediates **B6**, formed by coordination of the diazo species **8**, undergo subsequent insertion and protonation to access **B7**. Then, the product 1,2-benzothiazines **9** are generated by dehydration cyclization.

**Scheme 6:**
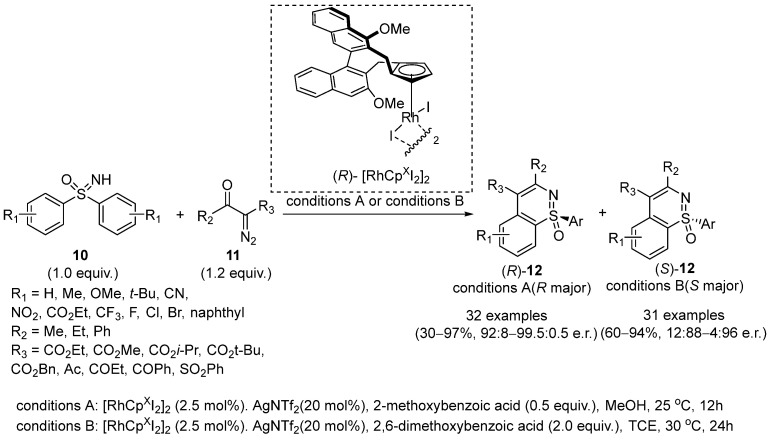
Rhodium-catalyzed strategy for the enantiodivergent desymmetrization of sulfoximines.

In 2018, Li and coworkers developed an enantiodivergent annulation between sulfoximines **10** and diazo compounds **11** catalyzed by rhodium(III) complexes [30]. In this strategy, the authors employed the Cramer type Cp*Rh(III) complexes and various carboxylic acids with different steric bulks to invert the absolute configuration of the product, which gave (*R*)-**12** and (*S*)-**12,** respectively, under the optimized conditions A and conditions B (Scheme 6).

**Scheme 7:**
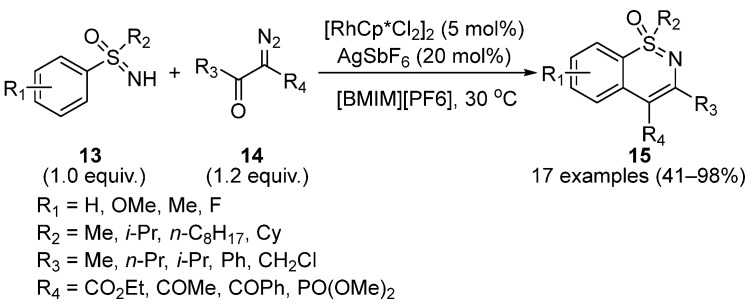
Sustainable Rh(III)-catalyzed C–H activation/cyclization of sulfoximines to form 1,2-benzothiazines.

One year later, Wang and Wu reported a sustainable Rh(III)-catalyzed C–H activation/cyclization of sulfoximines **13** with diazo derivatives **14** to produce 1,2-benzothiazines **15** in the ionic liquid [BMIM][PF_6_] (1-butyl-3-methylimidazolium-hexafluorophosphate) as a solvent (Scheme 7) [31]. This strategy exhibited a good tolerance for electron-donating and electron-withdrawing sulfoximine substituents. Moreover, this catalyst system could recycle at least 10 times before losing high-efficiency catalytic ability.

**Scheme 8:**
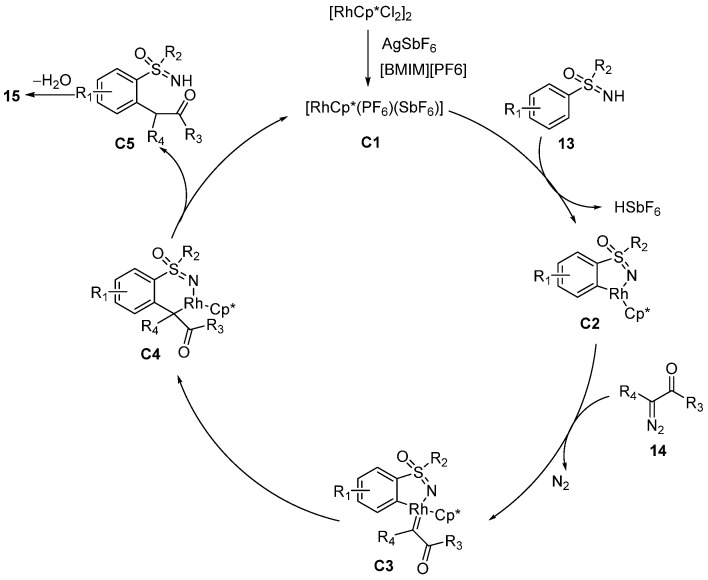
Proposed catalytic circle.

A proposed catalytic circle is shown in Scheme 8. In the beginning, the dissociation of the ionic liquid [BMIM] [PF_6_] gives PF_6_* as a substitute anion to activate the catalyst [RhCp*Cl_2_]_2_ in the presence of AgSbF_6_. Then, the activated catalyst **C1** is coordinated with sulfoximines **13** to form the five-membered Rh complexes **C2** and releases HSbF_6_. Subsequently, diazo derivatives **14** coordinate to the Rh center and afford the carbene species **C3**. Then, the insertion reaction of carbenes gives the six-membered intermediates **C4** and the following demetallization and intramolecular condensation furnish the product **15**.

**Scheme 9:**
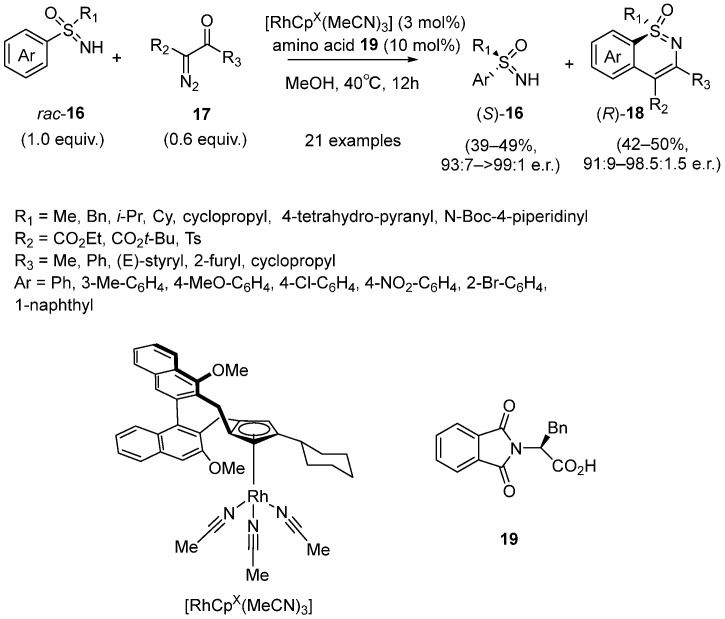
Rh(III)-catalyzed C–H functionalization strategy for the kinetic resolution of racemic sulfoximines.

In the same year, Cramer developed a Rh(III)-catalyzed C–H functionalization strategy for the kinetic resolution of racemic sulfoximines **16** to generate S-chiral 1,2-benzothiazines **18 [32]**. Compared to Cramer’s previous research in 2018, this approach could result in a higher e.r. value. In this case, sulfoximines were treated with diazoketoesters **17**, giving the desired products in high *s*-value (Scheme 9). Moreover, different substituted aryl sulfoximines could react smoothly and the enantioselectivity was up to >99:1, including the groups with remarkable steric hindrance, such as *i*-Pr and cyclohexyl. *Para*-nitrophenyl methyl sulfoximine was a supreme substrate under these conditions, resulting in s-values of more than 200 with 47% yields for both of the two products.

**Scheme 10:**
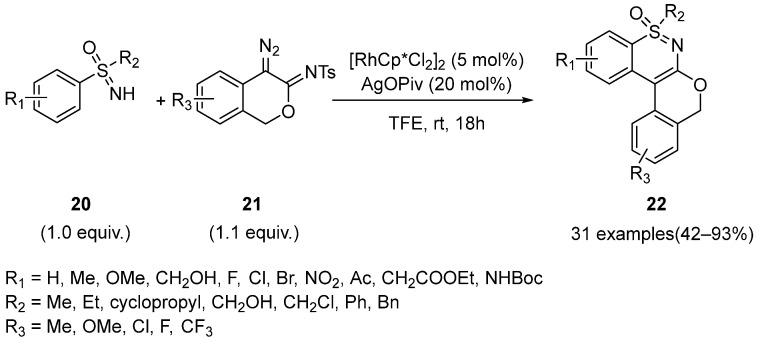
Rh(III)-catalyzed method for the synthesis of fused 1,2-benzothiazines from sulfoximines and 4-diazoisochroman-3-imines.

Highly fused polycyclic scaffolds exist widely in bioactive molecules, so their synthesis methods have also attracted much attention. In 2020, Li and Liu disclosed a Rh(III)-catalyzed method for the synthesis of fused 1,2-benzothiazines **22** from various sulfoximines **20** and 4-diazoisochroman-3-imines **21** (Scheme 10) [33]. Optimization studies showed that [RhCp*Cl_2_]_2_ (5 mol%) as a catalyst could work well with AgOPiv (20 mol%) as an additive under air in TFE at room temperature. In this strategy, haloalkanes were proved to be unfavorable to the yields because of the lack of polarity. Moreover, this reaction had a good substituent tolerance and resulted in moderate to excellent yields. However, *ortho*- and *para*-Me substrates could not form the desired products in high yields as expected, and the *meta*-Br substrate could give a single site-selective product while the *meta*-OMe substrate gave a mixture of two products.

**Scheme 11:**
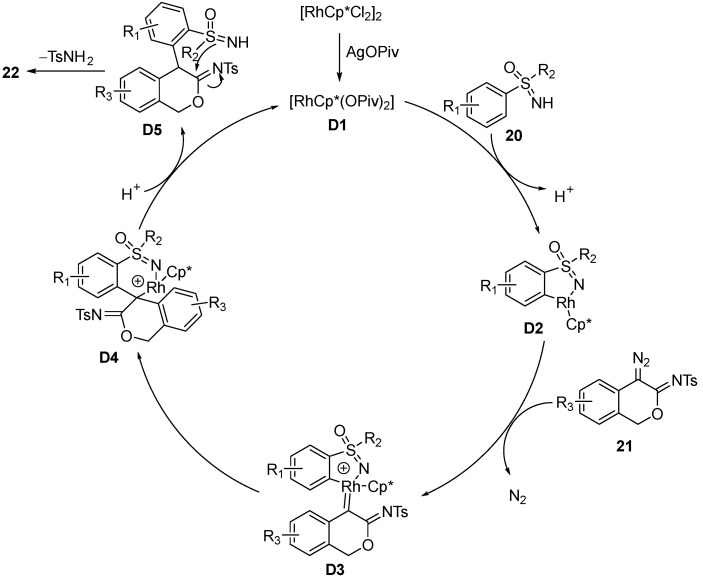
Proposed mechanism of the annulation between sulfoximines and 4-diazoisochroman-3-imines.

The mechanistic study demonstrated that the catalytic cycle (Scheme 11) begins with the formation of the rhodacycle intermediate **D2**, which is generated by the coordination of sulfoximine **20** to the Rh center of the activated catalyst [RhCp*(OPiv)_2_] **D1** and the electrophilic C–H bond cleavage. Next, the rhodium carbenoid complex **D3** is formed by the coordination of **D2** with diazo compound **21**, via the loss of nitrogen gas. Then, the insertion to the Ph–Rh bond gives intermediate **D4**, and subsequently, the catalyst is recycled by protonation of **D4**, giving intermediate **D5**. At last, the product **22** is obtained by the condensation of **D5** with the loss of TsNH_2_.

**Scheme 12:**
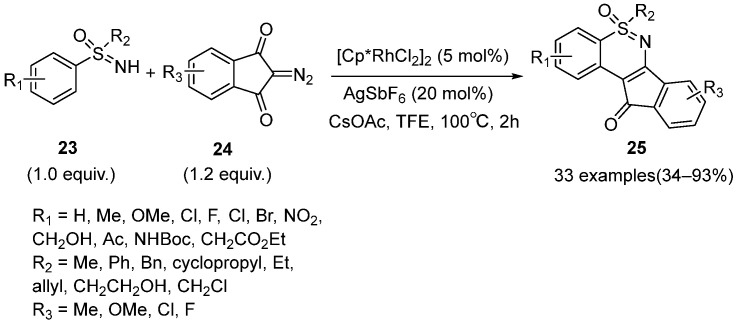
Rh-catalyzed synthesis of highly conjugated 1,2-benzothiazine scaffolds.

In 2021, Ye and Liu and coworkers described a methodology for the synthesis of highly conjugated 1,2-benzothiazine scaffolds **25** using sulfoximines **23** and 2-diazo-1H-indene-1,3(2H)-diones **24** (Scheme 12) [34]. The silver additive was proved to be important for high yields. Moreover, this protocol tolerated a wide range of substituted sulfoximines and provided target products with 74%–88% yields. Furthermore, it was worth mentioning that the C–H bond activation of *meta*-substituted sulfoximines tended to proceed on the more hindered *ortho*-positions. Various mono-substituted diazo compounds could also react smoothly, but the corresponding products were a regional isomers mixture.

**Scheme 13:**
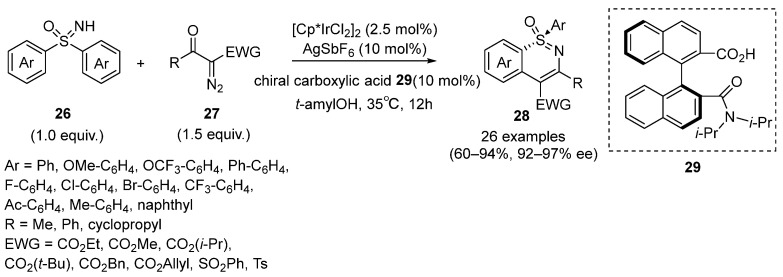
Ir(III)-catalyzed C–H activation/annulation strategy for the synthesis of chiral 1,2-benzothiazines.

Very recently, Hong and Shi reported an Ir(III)-catalyzed C–H activation/annulation strategy for the synthesis of chiral 1,2-benzothiazines **28** by the desymmetrization of sulfoximines **26** with diazo compounds **27** in the presence of chiral carboxylic acid **29** as ligand (Scheme 13) [35]. Various electron-withdrawing groups on the diazo compounds were screened to be well tolerated and the ee value was up to 97%. Both electron-donating and electron-withdrawing substituents of sulfoximines could also transform smoothly. Notably, the kinetic resolution and parallel kinetic resolution of racemic sulfoximines with this protocol were also developed by the authors.

#### 2.1.2. Ylide Active Intermediates as Carbene Precursors

**Scheme 14:**
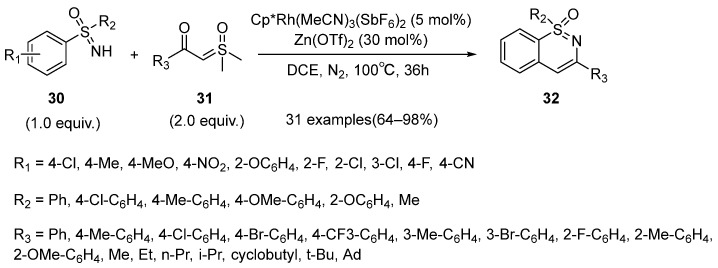
The synthesis of 1,2-benzothiazine through rhodium(III)-catalyzed annulative couplings.

In 2018, Li’s group reported a novel rhodium(III)-catalyzed annulative coupling strategy for the synthesis of 1,2-benzothiazines **32** between sulfoximines **30** and sulfoxonium ylides **31** (Scheme 14) [36]. Various substituted substrates were compatible. Moreover, different N-protected benz-amidines and benzophenone NH-imines were also tolerated, and they could provide various isoquinoline products in good yields. In order to clarify the reaction mechanism, a H/D exchange experiment was conducted and the results showed that partial H/D exchange was appeared at the *ortho*- position of the product. It was obvious evidence to reveal that the C–H activation is reversible. Moreover, the kinetic isotope effect of this reaction was also measured, and the result suggested that the C–H activation step was involved in the turnover-limiting process.

**Scheme 15:**
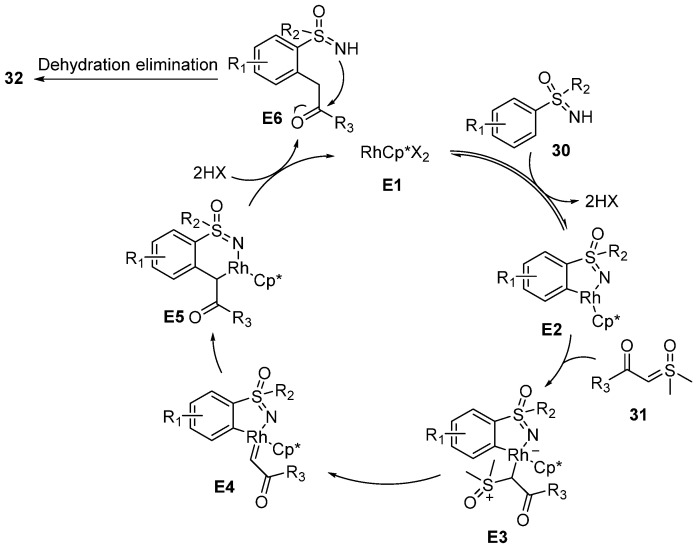
Proposed mechanism of the tandem annulative coupling between sulfoximines and sulfoxonium ylides.

The proposed catalytic mechanism is presented in Scheme 15. At first, the activated RhCp*X_2_ complex **E1** is coordinated with sulfoximine **30** to form intermediate **E2** by a metalation-deprotonation process. Then, the sulfoxonium ylides **31** coordinate to the rhodium center to generate intermediate **E3**. Subsequently, α-elimination of DMSO occurs, which leads to an active carbene complex **E4**. In addition, the Rh–Ar bond of the carbene species undergoes the migratory insertion pathway to generate intermediate **E5**. At last, reductive elimination and intramolecular dehydration obtain product **32**.

**Scheme 16:**
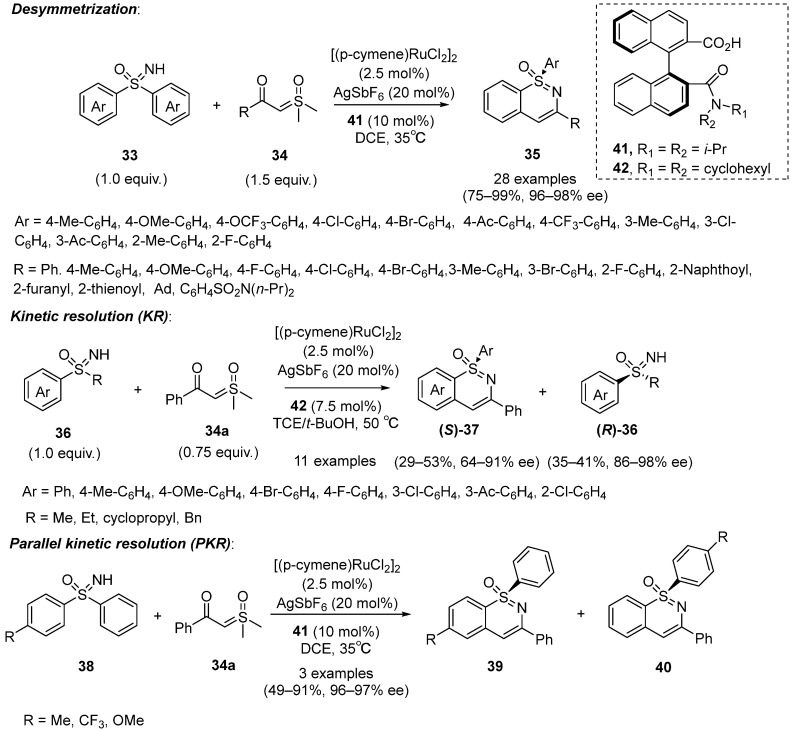
Desymmetrization, kinetic resolution, and parallel kinetic resolution of sulfoximines with sulfoxonium ylides under the Ru-catalyzed system.

In 2021, Shi’s group reported an enantioselective C–H functionalization of sulfoximines **33** with sulfoxonium ylides **34** using chiral binaphthyl carboxylic acids **41** as the optimal chiral ligand in combination with [(p-cymene)RuCl_2_]_2_ as a catalyst to obtain 1,2-benzothiazines **35** with good to excellent yields (75–99%) and excellent enantioselectivities (96–98% ee) (Scheme 16) [37]. Moreover, the kinetic resolution of racemic sulfoximines **36** could also be achieved when chiral binaphthyl carboxylic acids **42** were selected as the ligand, which produced the cyclization products (*S*)-**37** with low to moderate yields (29–53%) and moderate to excellent enantioselectivities (64–91%). Furthermore, the unreacted (*R*)-**36**, with low to moderate yields (35–41%) and excellent enantioselectivities (86–98%), could also be obtained. Fortunately, parallel kinetic resolution of racemic diaryl sulfoximine **38** could be carried out smoothly with high ee values (up to 95%) under the standard conditions.

**Scheme 17:**
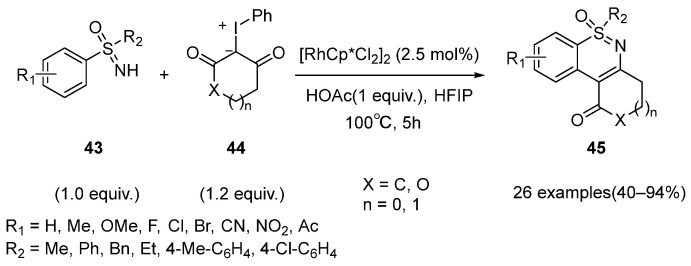
Rhodium-catalyzed strategy for the synthesis of polycyclic 1,2-benzothiazines from different aryl sulfoximines and iodonium ylides.

In 2021, Yu and Pan disclosed a rhodium-catalyzed strategy for the synthesis of polycyclic 1,2-benzothiazines **45** using different aryl sulfoximines **43** and iodonium ylides **44** as substrates (Scheme 17) [38]. Through the catalyst screening, [RhCp*Cl_2_]_2_ was proved to be the most efficient catalyst for this reaction system. Moreover, various polycyclic 1,2-benzothiazines with good functional group tolerance were furnished in moderate to excellent yields. 

**Scheme 18:**
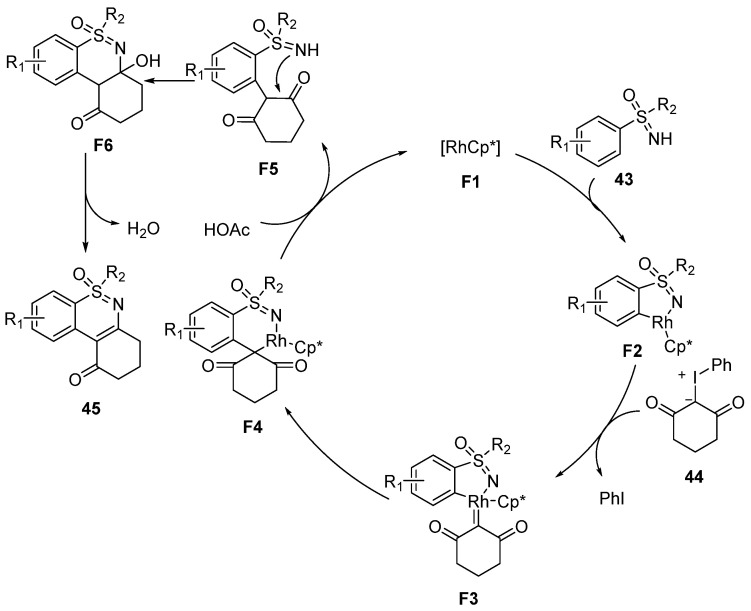
Proposed mechanism of the reaction between sulfoximines and iodonium ylides.

In order to reveal the reaction mechanism, the H/D exchange experiment and KIE studies were conducted. The results indicated that the C–H activation step may be irreversible and the cleavage of the C–H bond may not be involved in the rate-determining step. Moreover, the intermolecular competition experiments were also conducted and the results confirmed that electron-donating sulfoximines were beneficial to the C–H functionalization process. The proposed mechanism was similar to the previous reports (Scheme 18). At first, sulfoximines **43** coordinate to the Rh-catalysis **F1** to achieve C–H activation and yield a five-membered cyclic complex **F2**. Then, **44** reacts with **F2** to generate a carbene species **F3** along with the loss of PhI. Subsequently, the migratory insertion of Rh–carbene into the C–Rh bond is conducted, and obtains intermediate **F4**. After the reductive elimination of **F4**, the intramolecular nucleophilic cyclization and dehydration take place to form the desired product **45**.

**Scheme 19:**
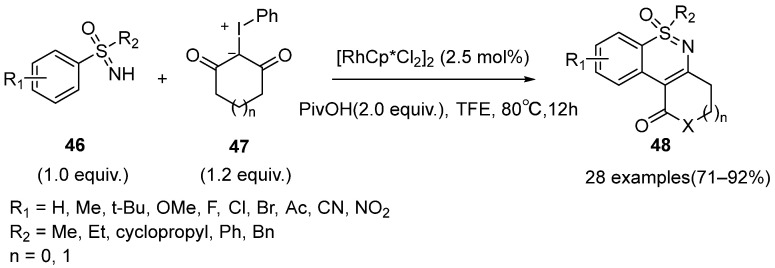
Rhodium-catalyzed strategy for the synthesis of polycyclic 1,2-benzothiazines.

Almost simultaneously, Wu et al. reported a very similar approach to provide polycyclic 1,2-benzothiazines **48** in good to excellent yields (Scheme 19). In this case, trimethylacetic acid (PivOH) was added as an additive [39]. Similar with Yu’s work, this protocol exhibited a broad substrate scope and was easy to obtain cyclohexanone-1,2-benzothiazine scaffolds. 

**Scheme 20:**
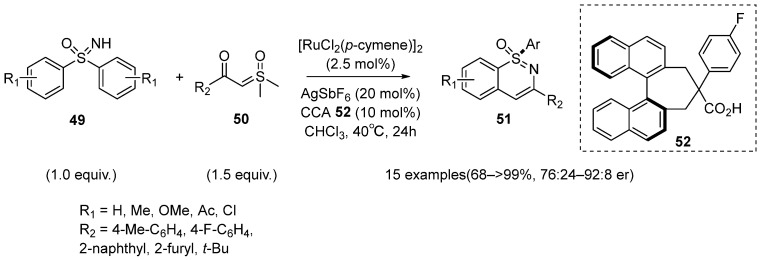
Ru(II)-catalyzed C–H functionalization strategy of sulfoximines for the enantioselective synthesis of 1,2-benzothiazines.

In 2022, Yoshino et al. developed a Ru(II)-catalyzed C–H activation/annulation of sulfoximines **49** with sulfoxonium ylides **50** for the enantioselective synthesis of 1,2-benzothiazines **51** (Scheme 20) [40]. The pseudo-C2-symmetric binaphthyl monocarboxylic acid **52** (10 mol%) proved to be the most optimal chiral ligand. A broad range of S-chiral 1,2-benzothiazine products were prepared in moderate to excellent yields with up to 92:8 er. Moreover, this protocol showed a compatibility for electron-donating substituents, electron-withdrawing substituents, and steric hindrance substituents.

**Scheme 21:**
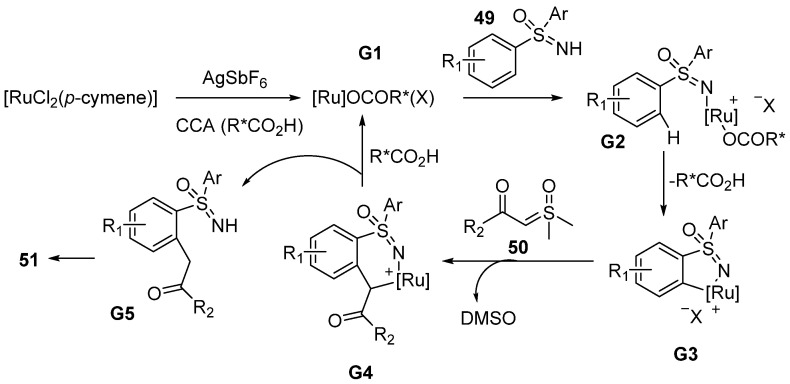
Proposed mechanism of Ru-catalyzed enantioselective synthesis of 1,2-benzothiazines.

A plausible catalytic cycle is outlined in Scheme 21. It includes five main steps. At the beginning, a Ru–carboxylate catalyst complex **G1** is formed by [RuCl_2_ (p-cymene)]_2_, AgSbF_6_, and **52**. Next, the sulfoximines **49** coordinate to **G1**, to assist C–H activation via a selective deprotonation process to give intermediate **G3**. Then, the chiral metallacycle complex **G3** coordinates with sulfoxonium ylides **50**, and next, the insertion of carbene to the Ru–Aryl bond gives a six-membered cycle **G4**, with the loss of DMSO in the reaction process. At last, the protonation and intramolecular condensation take place to access the desired products **51**.

### 2.2. Metal Nitrene as Coupling Partner

**Scheme 22:**
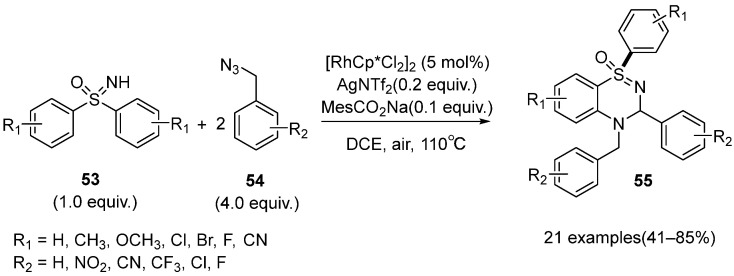
Rh(III)-catalyzed C–H functionalization of sulfoximines to prepare dihydrobenzo thiadiazine 1-oxide derivatives.

In 2019, Rh(III)-catalyzed C–H activation/cyclization for the synthesis of dihydrobenzo thiadiazine 1-oxide derivatives **55** using sulfoximines **53** and benzyl azides **54** as starting materials was reported by Xu and Dong (Scheme 22) [41]. This catalytic reaction was tolerant to various substrates bearing various electron-donating and electron-withdrawing groups, but the heterocyclic NH-sulfoximines and fluorinated or free benzyl azides afforded poor results. Additionally, the cyclization products were obtained in moderate to good yields (41–85%). 

**Scheme 23:**
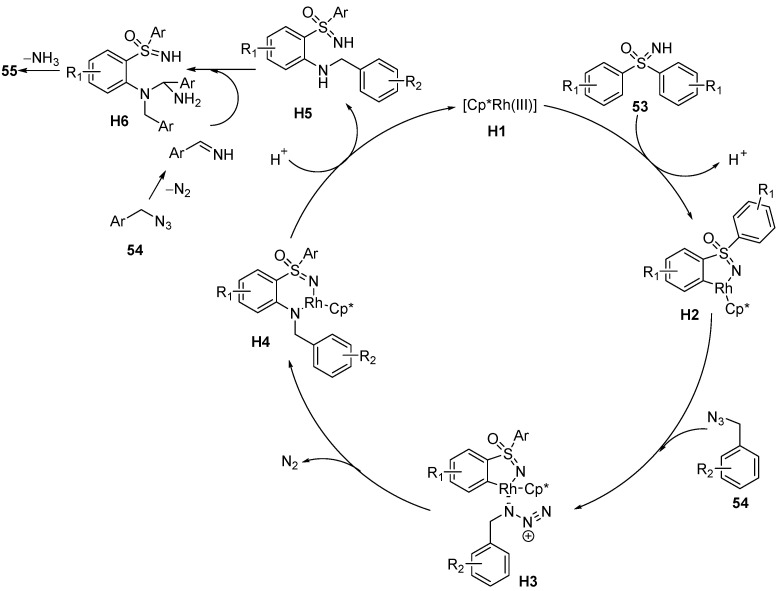
Proposed mechanism of C–H functionalization of sulfoximines and annulation with benzyl azides.

Based on a series of control experiments, a proposed mechanism is suggested in Scheme 23. Firstly, the sulfoximines **53** coordinate to the activated Rh(III) compound **H1** and subsequently undergo the C−H bond cleavage process to generate five-membered rhodacyclic complexes **H2**. After the coordination of the benzyl azides **54**, the nitrene intermediates **H3** are formed. Then, the migratory insertion occurs, and the C−N bond formation leads to a six-membered rhodium cycle **H4** with the loss of N_2_. Finally, protonolysis of **H4** leads to the formation of **H5** and recycle catalyst **H1**. Further cyclization leads to the formation of the observed products **55** with the loss of NH_3_.

**Scheme 24:**
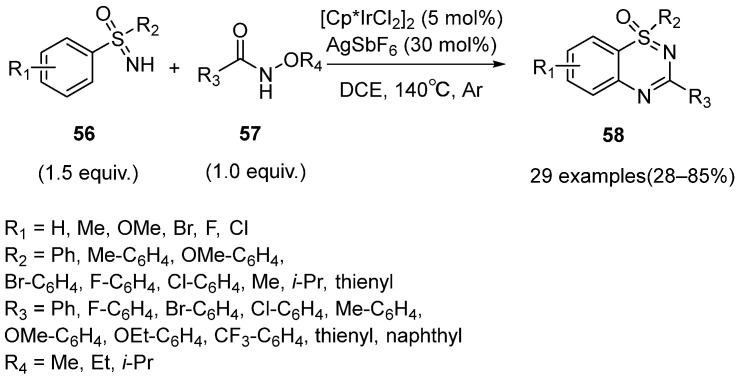
Ir(III)-catalyzed amidation/cyclization for the C–H activation of sulfoximines with N-alkoxyamides to synthesize the corresponding thiadiazine 1-oxides.

One year later, Dong’s group disclosed an Ir(III)-catalyzed amidation/cyclization of sulfoximines **56** with N-alkoxyamides **57** to synthesize thiadiazine 1-oxides **58** in low to high yields (28–85%) (Scheme 24) [42]. This method featured diverse substituents and functional groups tolerance, but the substrates with strong electron-withdrawing groups did not react smoothly.

**Scheme 25:**
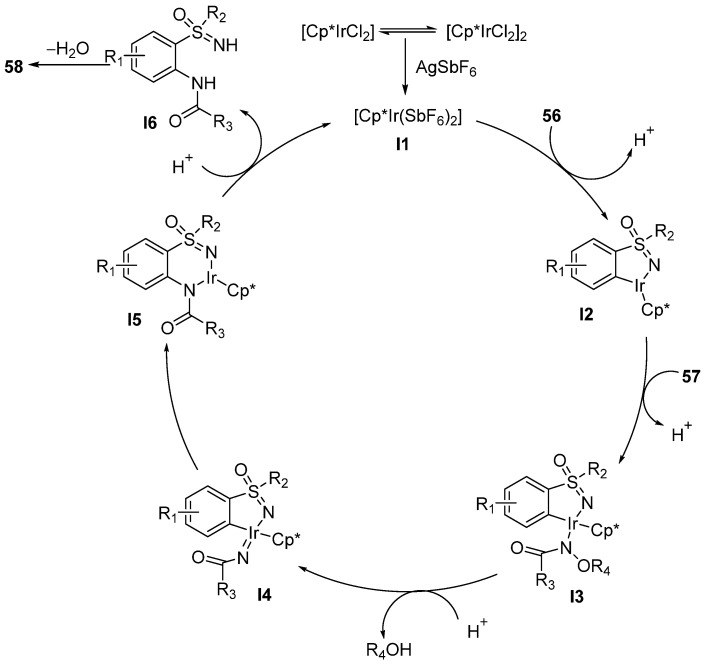
Proposed catalytic cycle.

According to the reaction mechanism (Scheme 25), the cleavage of the N–O bond of the intermediate **I3** was considered to be a key step, which generates the nitrene complex **I4** by protonation.

**Scheme 26:**
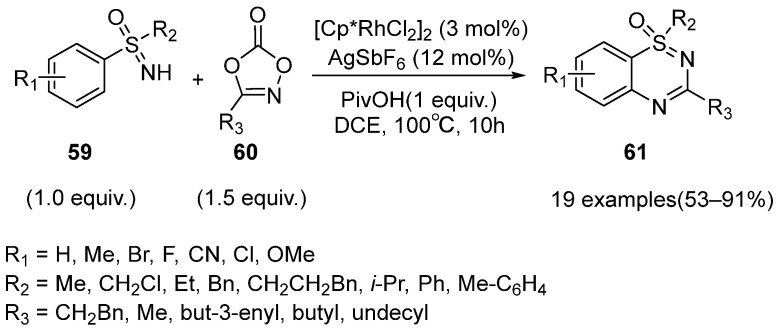
Rh(III)-catalyzed method to obtain benzothiadiazine-1-oxides using 1,4,2-dioxazol-5-ones.

In 2020, Bolm and coworkers reported an effective Rh(III)-catalyzed C–H functionalization cyclization strategy to prepare benzothiadiazine-1-oxides **61** from various sulfoximines **59** and 1,4,2-dioxazol-5-ones **60** (Scheme 26) [43]. This protocol could be conducted in the air without other protecting strategies. 

The results of the substrate scope studies show that all of the substrates with electron-donating, electron-withdrawing groups, and huge steric hindrance afforded the benzothiadiazine-1-oxides **61** in moderate to excellent yields (53–91%). Moreover, this work was well-suited for S-alkyl-S-aryl-substituted sulfoximines, which were specific comparing with the previous reports.

**Scheme 27:**
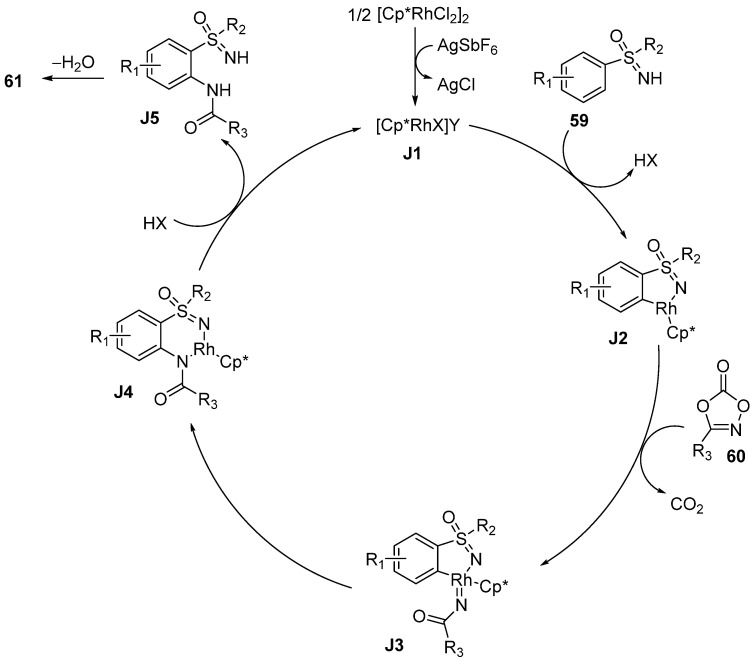
Proposed mechanism.

Consistent with the previously reported mechanism (Scheme 27), the five-membered rhodacycles **J2** are formed by the reaction of sulfoximines **59** and the activated Rh(III) catalyst. Then, they react with **60** to generate nitrenoid intermediates **J3**, accompanied by the loss of CO_2_. Next, the products **61** are obtained via a classical insertion–demetallization–condensation pathway.

**Scheme 28:**
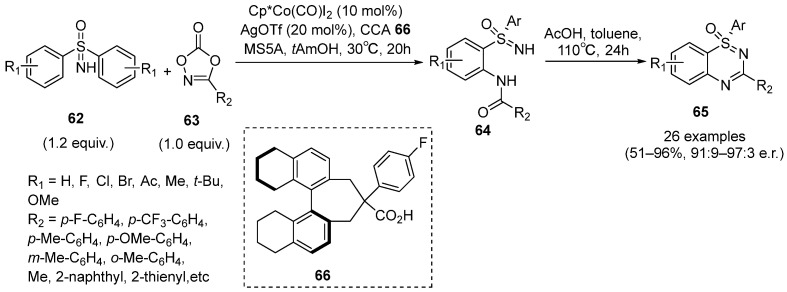
Co(III)-catalyzed enantioselective C–N bond formation of cyclic sulfoximines.

In 2022, Yoshino and coworker described a protocol involving Cp*Co(CO)I_2_/chiral carboxylic acid **66** as a catalytic system, which promoted the C–H functionalization reaction of sulfoximines **62** with dioxazolone **63** for the enantioselective synthesis of benzothiadiazine-1-oxides **65** (Scheme 28) [44]. This strategy consisted of two reaction steps. Firstly, under the transition metal catalyst system, sulfoximines **62** reacted with dioxazolones **63** to form the amidation product **64**. Then, **64** was treated with AcOH in toluene to give the cyclic products with moderate to excellent yields and good to excellent e.r. values. Moreover, the control experiments and DFT calculation were performed to confirm that the cleavage of the C–H bond is the enantiodetermining step. Furthermore, the studies of the scope of the substrates showed that this protocol has a good compatibility with several functional groups.

### 2.3. Alkenes as Coupling Partner

**Scheme 29:**
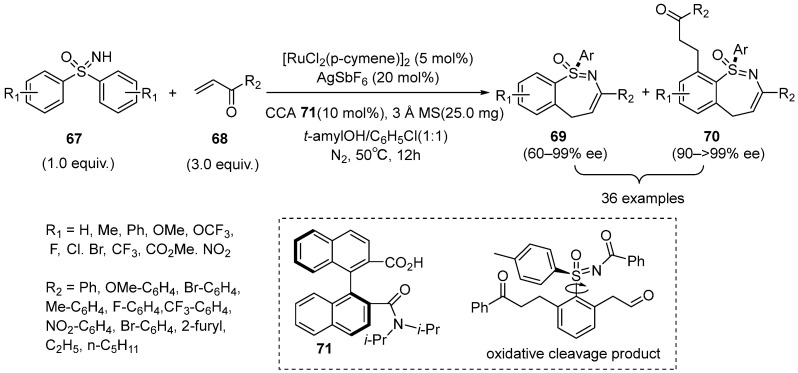
Ru(II)/chiral-carboxylic-acid-catalyzed enantioselectivity synthesis of seven-membered cyclic sulfoximines.

In 2022, Shi’s group developed an asymmetric [4+3] annulation between sulfoximines **67** and α,β-unsaturated ketones **68**, which was promoted by the Ruthenium(II) /chiral carboxylic acid **71** catalyst system. It generated a broad range of sulfur-stereogenic 1,2-benzothiazepine 1-oxides with up to 90% yield and up to >99% ee. It is worth noting that the positions of electron-withdrawing and electron-donating groups often led to the different main products. For example, *para*-substituted diarylsulfoximines bearing electron-donating groups (OMe, Ph) often benefited to the dialkylation/cyclization products, and the *para*- or *meta*-substituted diarylsulfoximines sulfoximines with electron-withdrawing groups would help to access mono alkylation/cyclization products. Moreover, a series of chiral N-benzoyl sulfoximines with a C–S chiral axis could be obtained after the oxidative cleavage of the double bonds in the products (Scheme 29) [45]. 

### 2.4. Alkynes as Coupling Partner

**Scheme 30:**
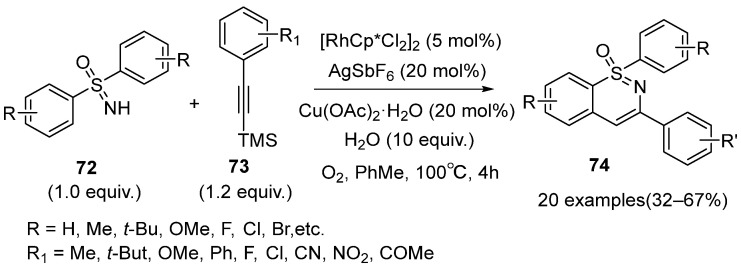
The synthesis of 1, 2-benzothiazines in the Rh(Ⅲ) catalytic system using alkynyl silanes.

It was generally considered that terminal alkynes were incompatible with the Rh(Ⅲ) catalytic system because of the existence of the active hydrogen atoms. In 2019, Prabhu et al. reported a novel strategy for the synthesis of 1,2-benzothiazines **74** using diphenyl sulfoximines **72** and alkynyl silanes **73** as substrates catalyzed by the Rh(Ⅲ) catalytic system (Scheme 30) [46]. In the presence of AgSbF_6_ and H_2_O, the TMS group was facilely removed from the alkynyl silanes to give the desired motifs. Oxygen was necessary for this reaction. A series of 1,2-benzothiazine derivatives with a free substituent at the C-4 position were obtained in low to moderate yields (32–67%), which had the potential to transform into other functional scaffolds. In addition, the degradation of the sulfoximines may be the reason for the relatively low yields.

**Scheme 31:**
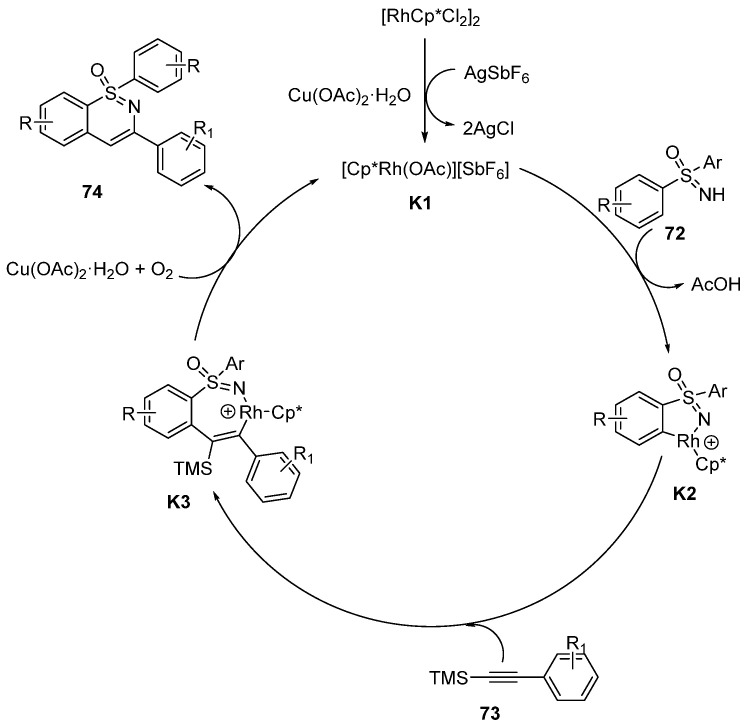
Proposed mechanism of annulation of sulfoximines and alkynyl silanes in the Rh catalytic system.

The plausible mechanism is demonstrated in Scheme 31. In the beginning of the cycle, AgSbF_6_ and Cu(OAc)_2_·H_2_O play a key role for the activation of the catalyst. Next, **K1** coordinates with sulfoximines **72** to form the five-membered rhodacycle complexes **K2**. Then, the insertion of arylalkynyl silanes **73** provides the intermediates **K3**. At last, the products **74** are obtained by reductive elimination of **K3**, and the active catalyst **K1** is also regenerated.

**Scheme 32:**
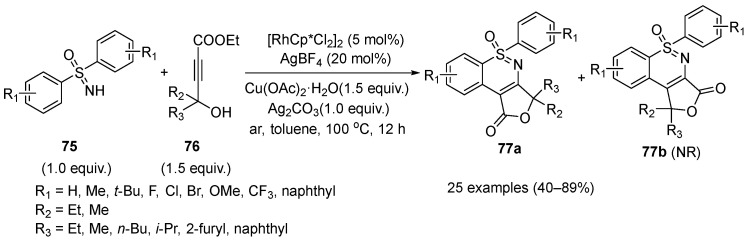
Tandem Rh(Ⅲ)-catalyzed C–H activation of sulfoximines to furnish furanone-fused 1,2-benzothiazines derivatives.

In the same year, Prabhu developed a Rh(Ⅲ)-catalyzed cascade C−H activation, regioselective annulation, and lactonization strategy for the synthesis of furanone-fused 1,2-benzothiazines derivatives **77a** using sulfoximines **75** and 4-hydroxy-2-alkynoates **76** (Scheme 32) [47]. The reaction exhibited good functional group tolerance and displayed regioselectivity in forming single regioisomers **77a** in moderate to good yields. The other regioisomer **77b** was not detected, which may be due to the steric interaction between the hydroxyl oxygen atom and the Rh(III) center. 

**Scheme 33:**
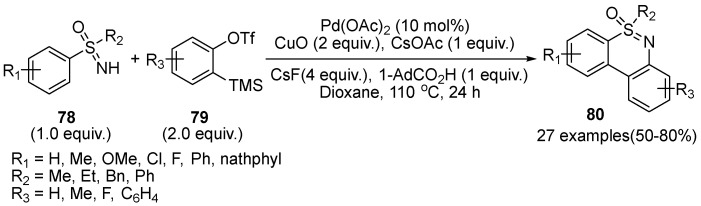
Pd(II)-catalyzed C–H functionalization strategy for the preparation of dibenzothiazines.

In 2020, Li et al. demonstrated Pd(II)-catalyzed C–H functionalization/cyclization of sulfoximines **78** with aryne precursors **79** to prepare tricyclic dibenzothiazines **80** by utilizing Pd(OAc)_2_ as the optimal catalyst, and CuO as the oxidant (Scheme 33) [48]. A variety of sulfoximines bearing electron-rich, halogen, and S-substituents performed well and the naphthyl- sulfoximine afforded a mixture of regioselective products.

**Scheme 34:**
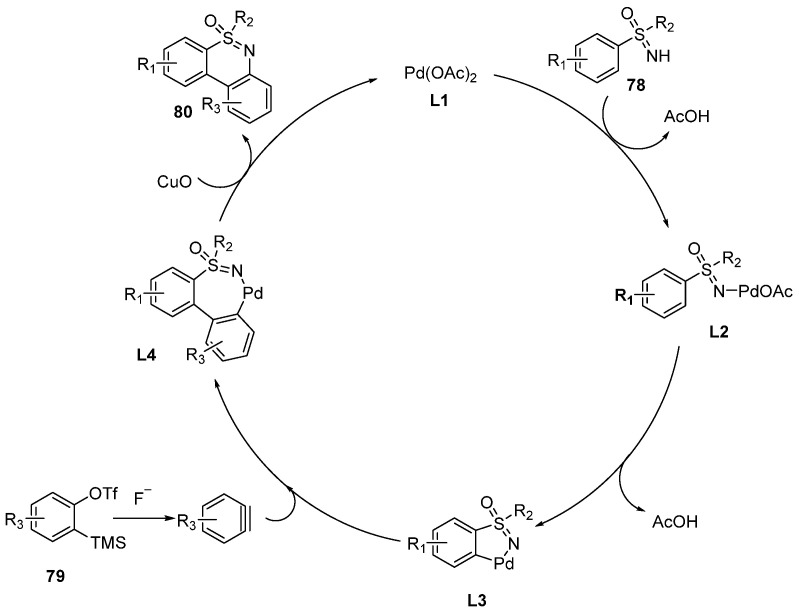
Proposed mechanism of Pd-catalyzed annulation of sulfoximines with arynes.

The proposed mechanism is shown in Scheme 34. Initially, Pd(OAc)_2_ reacts with sulfoximines **78** directly to generate the acyclic Pd species **L2** accompanied by the release of AcOH. Then, five-membered palladium cyclic complexes **L3** are formed by the intramolecular deprotonation with the loss of another molecule of AcOH. Subsequently, benzyne insertion occurs and produces seven-membered cyclic palladium complexes **L4**. At last, **L4** undergo a reductive elimination pathway to furnish the products **80**. Simultaneously, the activated catalyst **L1** is regenerated by reoxidation of Pd(0) to Pd(II) in the presence of the copper salt.

## 3. Synthesis of Cyclic Sulfoximines via Intramolecular C–H Activation

**Scheme 35:**
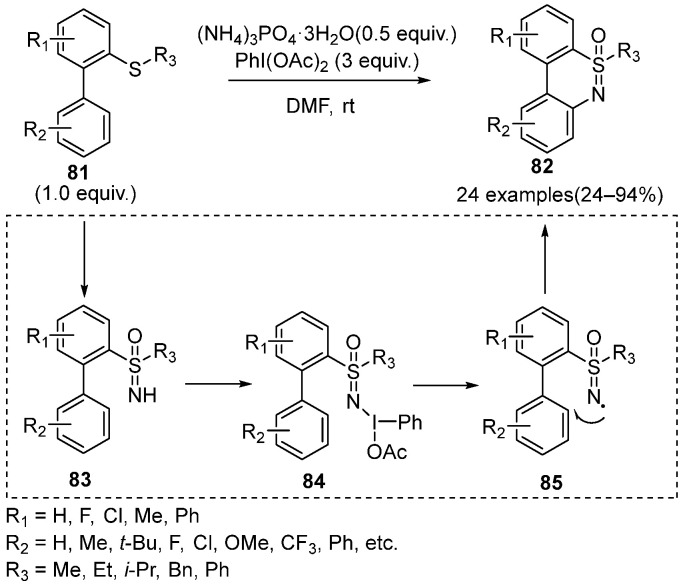
Metal-free method for the intramolecular C–N coupling to form benzothiazines.

In 2018, Zhang et al. reported a metal-free method to synthesize benzothiazines **82** through one-pot N,O-transfer and intramolecular C–H amination of 2-biphenylsulfoximines **81** (Scheme 35) [49]. The results of reaction conditions screening showed that selecting (NH_4_)_3_PO_4_·3H_2_O as the N-source and PhI(OAc)_2_ as the oxidant was necessary to generate the products **82** in high yields. Noteworthy, the mechanism studies proposed that PhI(OAc)_2_ could perform as a free radical initiator for NH-sulfoximines to form the crucial intermediate **84** and **85** in the plausible pathway. Moreover, this protocol has a wider universality to the substrates. Various dibenzothiazine derivatives could be conveniently accessed in the yield ranging from 24–94%.

**Scheme 36:**
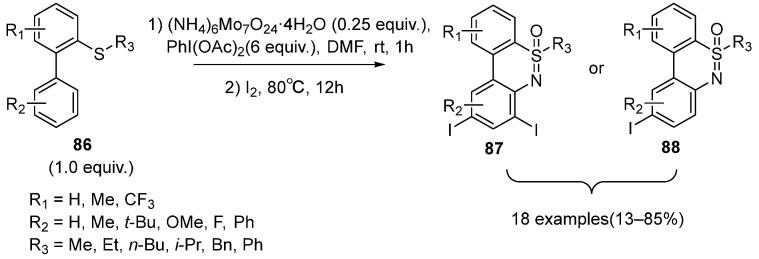
Tandem C–H activation and C–N coupling of sulfoximines to form iodo-thiazines.

Based on the previous studies, Zhang’s group further investigated the one-pot N,O-transfer and intramolecular C–H amination of 2-biaryl sulfides **86** to afford a series of iodo-dibenzothiazines compounds **87** (Scheme 36) [50]. However, monoiodo-dibenzothiazines **88** were also observed when the 4′-positions of sulfides were substituted by large steric hindrance substituents or the strong electron-withdrawing substituents such as CF_3_. Moreover, the desired products could be further transformed to other functional thiazine compounds through the cross-coupling reaction. 

**Scheme 37:**
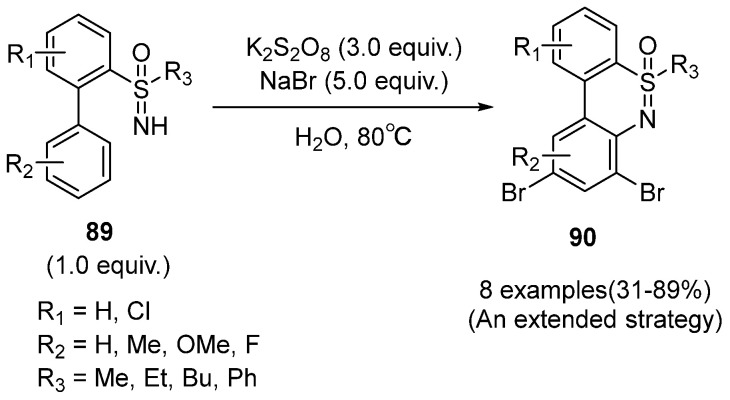
Metal-free tandem amination/bromination method for the synthesis of bromobenzothiazines.

In 2019, Ma and coworkers reported a metal-free tandem amination/bromination method for the synthesis of bromobenzothiazines **90** by intramolecular C–H activation of N-methyl-2-biphenylsulfonamides and 2-biphenylsulfoximines **89** (Scheme 37) [51]. Interestingly, different from the sulfonamides substrates, sulfoximines could afford dibromothiazines as the only products and the momo-bromothiazines were not detected under the standard conditions.

**Scheme 38:**
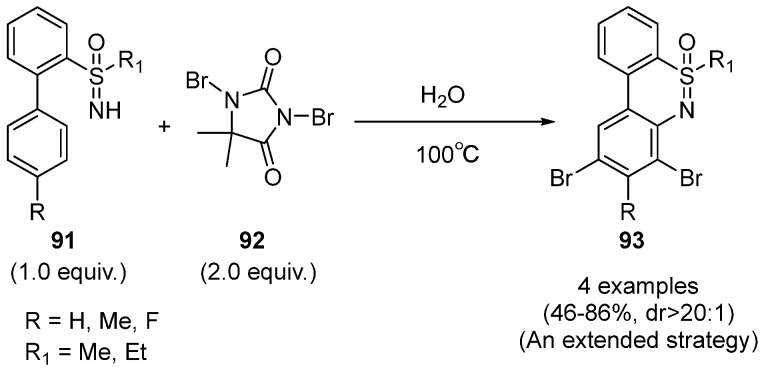
DBH-promoted strategy for the cyclization of sulfoximines.

One year later, Zhang and Chen developed a step-economic DBH-promoted cyclization strategy for the synthesis of bromo-N-heterocycles **93** from the 2-biphenyl sulfoximines **91** in water (Scheme 38) [52]. Moreover, this novel strategy fulfilled the synthesis of various N-heterocycles products in moderate to good yields and up to >20:1 dr, and the substrate scope was also extended to 2-biphenyl phosphamides.

## 4. Miscellaneous

**Scheme 39:**
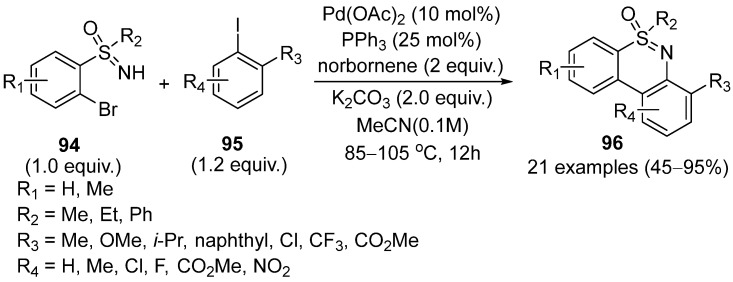
Pd/norbornene-catalyzed coupling reaction for the synthesis of fused polycyclic sulfoximines.

In 2018, Chen and coworkers reported the Pd/norbornene-catalyzed coupling reaction for the synthesis of fused polycyclic sulfoximines **96** with 2-bromo-NH-sulfoximines **94** and aryl iodides **95** (Scheme 39) [53]. PPh_3_ was confirmed to be the optimal ligand and norbornene was proved to be necessary for this reaction. The scope of substrates was also explored under the optimized conditions. Reaction of aryl iodides and ortho-bromophenyl sulfoximines successfully afforded the desired products in good yields. Moreover, aryl iodides with electron-withdrawing groups (CO_2_Me and CF_3_) also transformed smoothly. However, *ortho*-substituent would affect the reaction process. 

## 5. Conclusions

A number of protocols reported in the recent few years for the synthesis of cyclic sulfoximines via C–H bond activation have been summarized in this review. Various coupling partners including diazo compounds, sulfoxonium ylides, iodonium ylides, benzyl azides, N-alkoxyamides, 1,4,2-dioxazol-5-ones, α,β-unsaturated ketones, alkynyl silanes, 4-hydroxy-2-alkynoates, and arynes were successfully assembled with NH-sulfoximines, forming the corresponding [4+2] or [4+3] annulation products bearing a variety of substituents. Despite the huge advances in this area, there still remain a number of limitations. The commonly used catalysts such as Ru(II) and Rh(III) are costly, while the relatively cheap Co(III) catalysts have not been employed widely or performed poorly. The metal-free methods are still limited to intramolecular reactions and the scope of substrates is generally narrow. Except the nitrogen atom, it has not been reported that other heteroatoms are introduced into the cyclic sulfoximines. Furthermore, the cyclic products formed are almost six- and seven-membered sulfoximines. We hope that this review will inspire more researchers to conceive more fantastic ideas and to find more valuable new synthetic methods.

## Data Availability

No new data were created.
